# CONVOYS OF CARE

**DOI:** 10.1093/geroni/igac059.1706

**Published:** 2022-12-20

**Authors:** Toni Antonucci, Kristine Ajrouch

**Affiliations:** University of Michigan, Ann Arbor, Michigan, United States; Eastern Michigan University, Ypsilnati, Michigan, United States

## Abstract

Many existing Alzheimer’s disease (AD) caregiving interventions focus narrowly on thechallenges and needs of a primary caregiver rather than the family systems in which they areembedded. We advance a family systems framework by invoking convoys of caregiving to adaptan existing AD caregiver intervention to Middle Eastern/Arab American families in metroDetroit (N=56). The composition of caregiving networks is described, followed by assessment ofcare burden, depressive symptoms, care satisfaction and family conflict. Results show thatsiblings and children are the predominate support network members who accompanied theprimary caregiver to the program. Paired t-tests show that care burden and family conflictdecreased while caregiving satisfaction increased following program participation. Depressivesymptoms did not change. Findings illuminate how convoys of care may serve as valuablesupport resources, yet may also be the source of stress and conflict.

